# Adsorption of Pb (II) and Zn (II) ions from aqueous solutions by Red Earth

**DOI:** 10.1016/j.mex.2020.100804

**Published:** 2020-01-23

**Authors:** Abbas Esmaeili, Hadi Eslami

**Affiliations:** a Occupational Environment Research Center, Department of Environmental Health Engineering, School of Health, Rafsanjan University of Medical Sciences, Rafsanjan, Iran; b Department of Environmental Health Engineering, School of Health, Rafsanjan University of Medical Science, Rafsanjan, Iran; c Pistachio Safety Research Center, Rafsanjan University of Medical Science, Rafsanjan, Iran

**Keywords:** Adsorption, Heavy metal removal, Lead, Zinc, Red Earth

## Abstract

This study was carried out to investigate Pb (II) and Zn (II) removal from aqueous solutions by Red Earth (RE) as a new local natural adsorbent in using the batch method. The chemical structure of RE adsorbent was characterized by XRF. Giles, Langmuir, and Freundlich isotherms were used to describe the adsorption data. The effect of metals concentration, initial pH, adsorbent dosage, and agitation time were studied. The results showed that RE contains of SiO_2_ (58 %) and Al_2_O_3_ (15.2 %) as major compounds. The equilibrium time was reached following 30 min and the maximum adsorption capacities (mg/g), based on Langmuir equation were 10.31 and 8.74 for Pb (II) and Zn (II), respectively. By increasing the initial metal ions concentration, the adsorption efficiencies were decreased and adsorption capacity of RE increased with an increase in the initial pH.

## Graphical abstract

**Figure:**
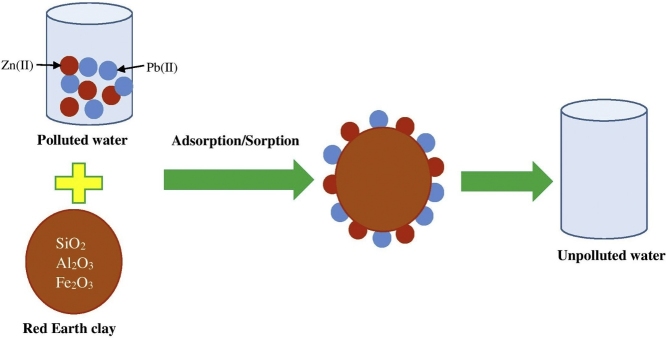

**Specification Table**

**Table:** 

Subject area:	Environmental Engineering, Chemical Engineering
More specific subject area:	Adsorption, Heavy metals removal
Protocol name:	Adsorption of Pb (II) and Zn (II) Ions from Aqueous Solutions by Red Earth (RE)
Reagents/tools:	A Native RE (from Iran) was used as an adsorbent and characterized by XRF. Also, Pb (II) and Zn (II) concentration were determined by atomic absorption spectroscope (AAS)
Experimental design:	Adsorption efficiency of RE adsorbent in various levels of initial metals concentration, initial pH, adsorbent dosage, and agitation time was tested.
Trial registration:	Not applicable
Ethics:	Not applicable

**Value of Protocol**

**Table:** 

• RE as a new local natural adsorbent was used for removal of Pb (II) and Zn (II) Ions from aqueous solutions • The maximum adsorption capacities (mg/g) of RE were 10.31 and 8.74 for Pb (II) and Zn (II), respectively (Pb^2+^>Zn^2+^). • It can be concluded that RE is a suitable adsorbent for removing Pb (II) and Zn (II) from contaminated water and wastewater.

## Description of protocol

### Preparation of adsorbent

The adsorbent of this series of tests was a local natural RE from Goud-E-Ahmar region in Kerman province located in the south east part of Iran. It was used for removal of Pb (II) and Zn (II) Ions from aqueous solutions as a new local natural adsorbent. First, RE was crushed using jaw crusher, then sieved and particles less than 50 mesh were selected for tests. Also, the adsorbent was dried for two weeks in the laboratory temperature [1,2]. Chemical and mineralogical composition of RE was determined by X-ray Florescent (XRF) (PHILIPS PW1730, Netherlands); shown in Table 1. The XRF analysis showed that RE contains SiO_2_ (58 %), Al_2_O_3_ (15.2 %) as major compounds.

**Table 1:** XRF analysis of dried Red Earth.

Composition	Wt%
SiO_2_	58.0
Al_2_O_3_	15.2
Fe_2_O_3_	3.1
CaO	4.7
MgO	0.8
Na_2_O	Tr.
K_2_O	0.1
LOI^a^	10.7

a LOI: Loss of Ignition.

### Experiment procedure

Solutions and reagent used in this study were all analytical grade reagents which were prepared from E-Merck (with ≥99 % purity). Heavy metal solutions were prepared from nitrate salts at a concentration of 1000 mg/L as a stock solution. Stock solution was stored in bottles with tight lid and other required solutions were prepared from the solution. For measuring and adjusting pH, the YSI portable (HQ 40d, Hach, Germany) and NaOH and HNO_3_ with concentration of 1.0 N were used. Batch experiments were applied for Pb(II) and Zn(II) ions adsorption from aqueous solutions, as a single metal system. In this study, the following conditions were constant in all tests; temperature (27 ± 1 °C), particle size (50 mesh), solution volume (50 mL), and shaking rate (150 rpm). For determining metals ions concentration in all solutions, atomic absorption spectroscopy (AAS) methods were used (Varian AA-975 and AA-1275 models).

The percentage of metal ions removal by RE was calculated by the following equation (Eq. (1)):

(1) $$\%RE=\frac{C_{O}-C_{e}}{C_{O}}*100$$

The adsorption capacity of RE was calculated by applying the mass balance equation (Eq. (2)):

(2) $$\text{q}\text{e}=\frac{C_{O}-C_{e}}{m}*V$$

In which, q_e_ is amount of metal ions absorbed on RE (mg/g); C_0_ and C_e_ are the initial and equilibrium concentration (mg/L) of Pb (II) and Zn (II) ions, respectively; V is the volume of meal ions solution (L) and m is mass of adsorbent used (g) [1].

### Adsorption isotherms

**Fig. 1:**
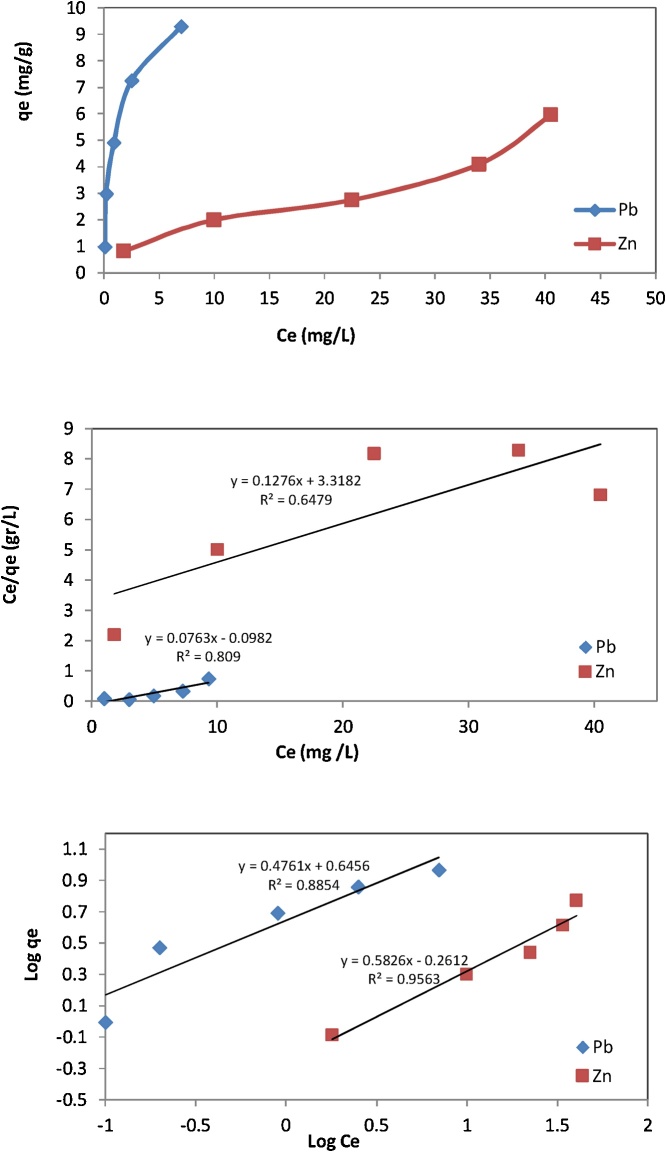
Giles classification (a), Langmuir (b) and Freundlich (c) adsorption isotherms of Pb^2+^ and Zn^2+^.

Isotherm equations are used for describing adsorption data for wide range of adsorbate concentrations. The most important isotherm equations are Langmuir and Freundlich [3,4]. Also, in some studies, Giles classification isotherms which is a qualitative descriptive model is used to describe the adsorption data [1]. Giles classification, Langmuir and Freundlich isotherms are shown in Fig. 1. The results showed that adsorption of Zn^2+^ and Pb^2+^ on RE were fitted by Freundlich (Fig. 1b) and Langmuir (Fig. 1c) isotherm models, respectively. However, satisfactory correlation coefficients were obtained using later equations for Zn^2+^ R^2^>0.98 and, Pb^2+^R^2^>0.99. Moreover, maximum adsorption capacities Q_0_ (mg/g), based on Langmuir equation were 10.31 and 8.74 for Pb^2+^ and Zn^2+^, respectively (Table 2).

**Table 2:** Freundlich and Langmuir constants.

	Freundlich adsorption			Langmuir adsorption		
	k_f_	N	R^2^	Q_0_	b	R^2^
Pb^2+^	4.43	2.11	0.88	10.31	1.18	0.81
Zn^2+^	0.55	1.72	0.95	8.74	0.04	0.64

### Effect of initial metals concentration

**Fig. 2:**
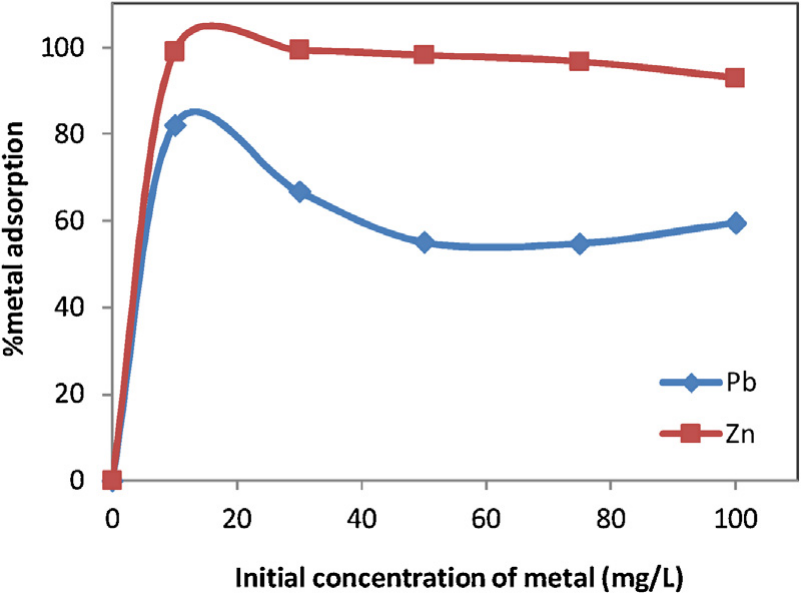
Effect of initial concentration on the sorption of Pb^2+^ and Zn^2+^ ions by RE.

For determining metals concentration, Pb(II) and Zn(II) ions were investigated by adding 0.5 g of RE with 50 ml of meal ions of Pb(II) and Zn(II) solutions at concentrations of 10, 30, 50, 75, and 100 mg/L. The pH of solutions was adjusted in 3.0 ± 0.1 with NaOH and HNO_3_ concentrations of 1.0 N, then the suspension was transferred in rotary shaker and shaken for 300 min with speed of 150 rpm. After reaching equilibrium, suspensions were filtered using Whatman filter paper and then metals ions concentration in filtrate was determined as the amount of removal. The effect of initial metals concentration on the removal of Pb(II) and Zn(II) ions from aqueous solutions by RE is shown in Fig. 2. As the results show, by increasing initial concentration of Pb(II) and Zn(II)ions, the removal percentage decreases with gentle slopes; however, increasing the amount absorbed ions per unit weight of the adsorbent (mg/g) was occurred by increasing in initial metal concentration [5].

### Effect of adsorbent dosage

**Fig. 3:**
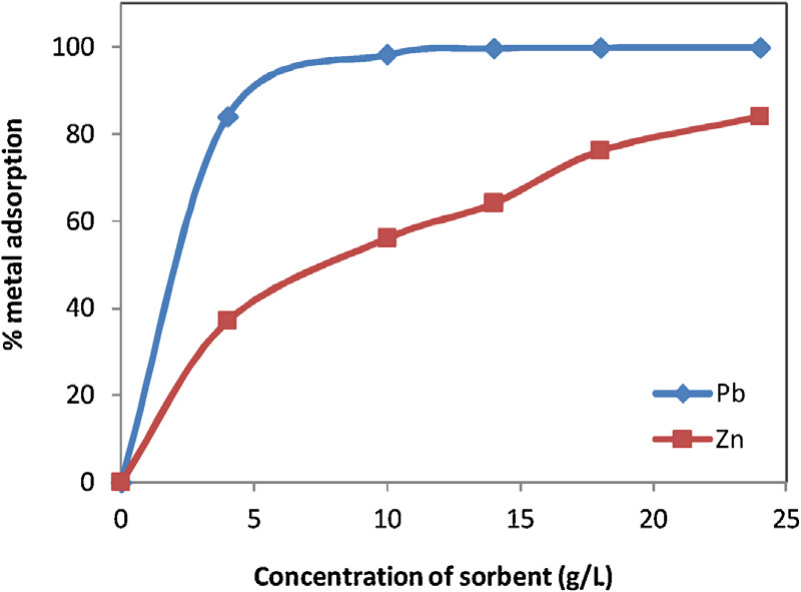
Effect of concentration on the sorption of Pb^2+^ and Zn^2+^ ions by RE.

The dosage of RE adsorbent varied from 4−24 g/L with a constant concentration of Pb(II) and Zn(II) ions (50 mg/L). Then solution volume (50 mL) mixed in 300 mL flat bottom Erlenmeyer flasks was moved to rotary shaker and shaken for 300 min with speed of 150 rpm. The suspensions were filtered using Wathman filter paper and then metal ions concentration in filtrate was determined as the amount of adsorbent. The effect of adsorbent dosage is shown in Fig. 3. As can be observed, increasing RE dose increases adsorption of Pb (II) and Zn (II) ions. Removal percentage of Pb (II) and Zn (II) ions at a dose of 24 g/L of RE is 99.9 % and 84 %, respectively. In fact, two mechanisms occur by increasing adsorbent amount. The first mechanism is increasing the surface area for ions adsorption and the second is increasing final pH of solutions by increasing the amount of adsorbent ; therefore, heavy metals ions were precipitated [6,7].

### Effect of initial pH

**Fig. 4:**
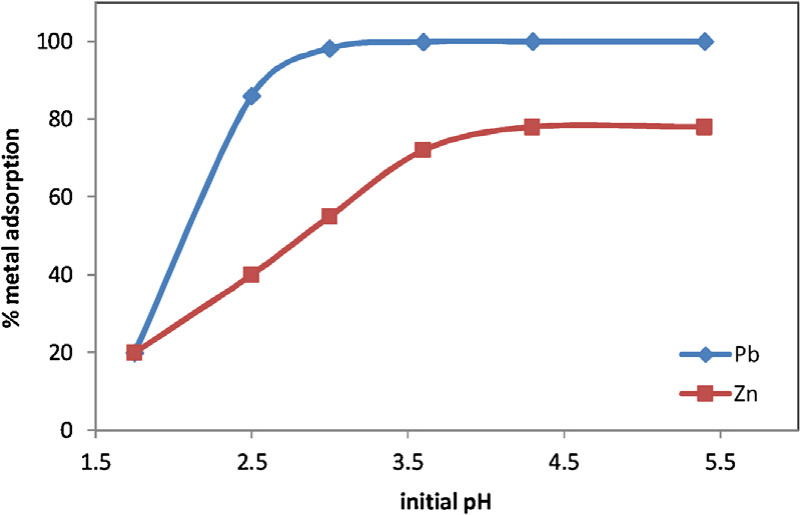
Effect of initial pH on the sorption of Pb^2+^ and Zn^2+^ ions by RE.

For determining the pH effect on adsorption capacity of Pb(II) and Zn(II) ions from solutions by RE, first 50 ml of Pb (II) and Zn (II) solutions (50 ppm) was mixed with 0.5 g of RE adsorbent and then the pH of solutions was adjusted in the range of 1.75–5.5 to prevent chemical precipitation phenomena and to guarantee that removal of heavy metal ions are essentially due to the adsorption method, pH was selected below 7.0 level). The effect of initial pH of solutions is shown in Fig. 4. The results show that at low pH the adsorption is negligible; however, the percentage of absorption of Pb (II) and Zn (II) ions increases by increasing pH. It is a common finding for adsorption of metal ions by natural clay minerals. In strongly acidic solutions, the number of H^+^ ions are predominant and compete with metals ions uptake by active sites on the adsorbent. Consequently, the removal of Pb(II) and Zn(II) ions from solutions by RE is less. By increasing pH the amount of H^+^ ions decreases and then the competition between H^+^ ions and metal ions for adsorption sites decreases. Thus, removal of Pb (II) and Zn (II) ions by RE increased [5,8,9]. In between pH values 2.5 and 3.5, these percentages increase sharply, attaining values that stay almost constant for higher pH values. In addition, increasing initial pH of solutions increases final pH of solutions; therefore, precipitation of heavy metals ions maybe occurs. Therefore, metal ions removal occurs via several mechanisms, such as chemical precipitation, ion exchange, complex formation, and co-precipitation [10].

### Effect of agitation time

**Fig. 5:**
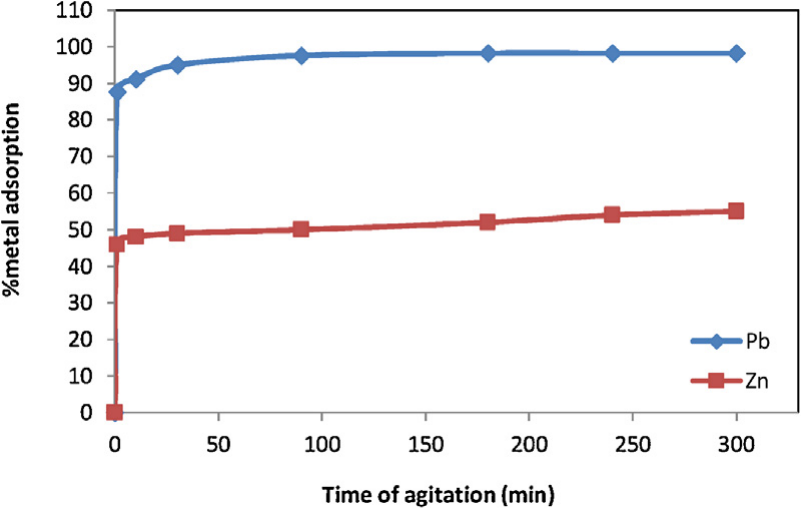
Effect of agitation time on the sorption of Pb^2+^ and Zn^2+^ ions by RE.

50 ml of Pb (II) and Zn (II) solutions (50 ppm) were mixed with 0.5 g of RE adsorbent. The pH of solutions was adjusted at 3.0 ± 0.1. Then the suspension was moved in rotary shaker and shaken for 1−300 min at speed of 150 rpm. The suspensions were then filtered using Whatman filter paper and metals concentration in filtrate was determined as the amount of adsorbent. Fig. 5 shows the effect of contact time on the removal of Pb(II) and Zn(II) ions from aqueous solutions by RE. As it can be seen the time to reach equilibrium is quick (30 min), indicating that the adsorption sites are well exposed.
